# Novel ADMET design tool for chemists

**DOI:** 10.1186/1758-2946-3-S1-P9

**Published:** 2011-04-19

**Authors:** DW Miller, R Fraczkiewicz, WS Woltosz

**Affiliations:** 1 Simulations Plus, Inc., Lancaster, CA, USA

## 

We present a new tool for molecule design called ADMET Sketcher™. The tool allows chemists to draw any number of molecules inside of a canvas, and immediately obtain predicted values of dozens of key ADMET properties from our best-in-class ADMET Predictor™ software package. The sketcher itself includes a number of novel capabilities, including a feature to modify both the bond angles and torsional angles of molecule side chains, and an advanced structure-cleanup feature with the option to keep one or more regions of the molecule fixed in place. Yet the true power of the tool comes from the numerous ADMET predictions covering physicochemical, biopharmaceutical, metabolism, and toxicities, which are updated dynamically as structures are edited, allowing near instantaneous feedback about which structural changes affect which properties, and in what manner. The ability to handle multiple structures means that a molecule found to have a desirable property profile can be kept as a reference, so properties of subsequently drawn molecules can quickly and easily be compared with the original. This is particularly useful when designing analogs of a known lead. We illustrate the tool by examining a diverse set of structural changes within a collection of molecules active against the HIV Integrase enzyme.

